# Supplementary figures and images for: DNA-PK promotes DNA end resection at DNA double strand breaks in G0 cells
Source: eLife. 2022 May 16;11:e74700. doi: 10.7554/eLife.74700 (PMC9122494; doi:10.7554/eLife.74700)

9/12/14

Lj4-45.  
P3-M69-169.16

|       | Lj4-45.<br>P3-M69-169.16 |   |   |
|-------|--------------------------|---|---|
| gCtIP | -                        | + | - |
| Mre11 | -                        | - | + |

CtIP

CtIP

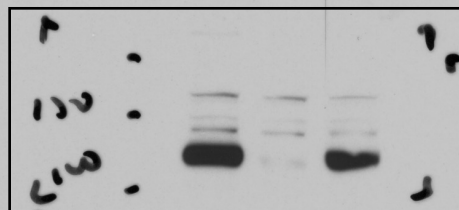

Mre11

Mre11

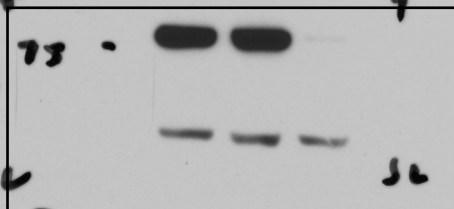

GAPDH

GAPDH

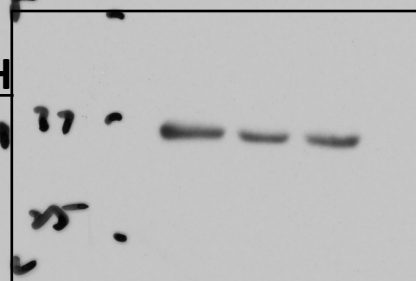

(5/19/10 samples)

Supplement: Figure 1—figure supplement 1—source data 1. [file elife-74700-fig1-figsupp1-data1.pdf]

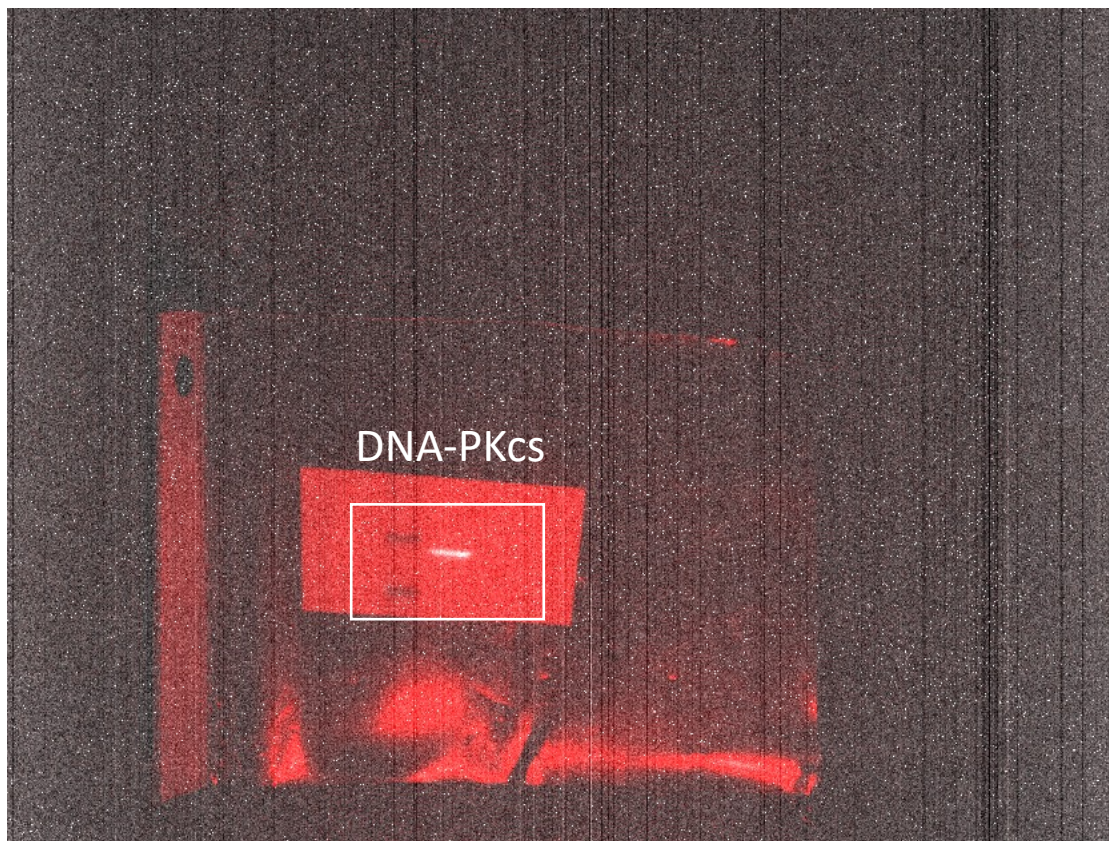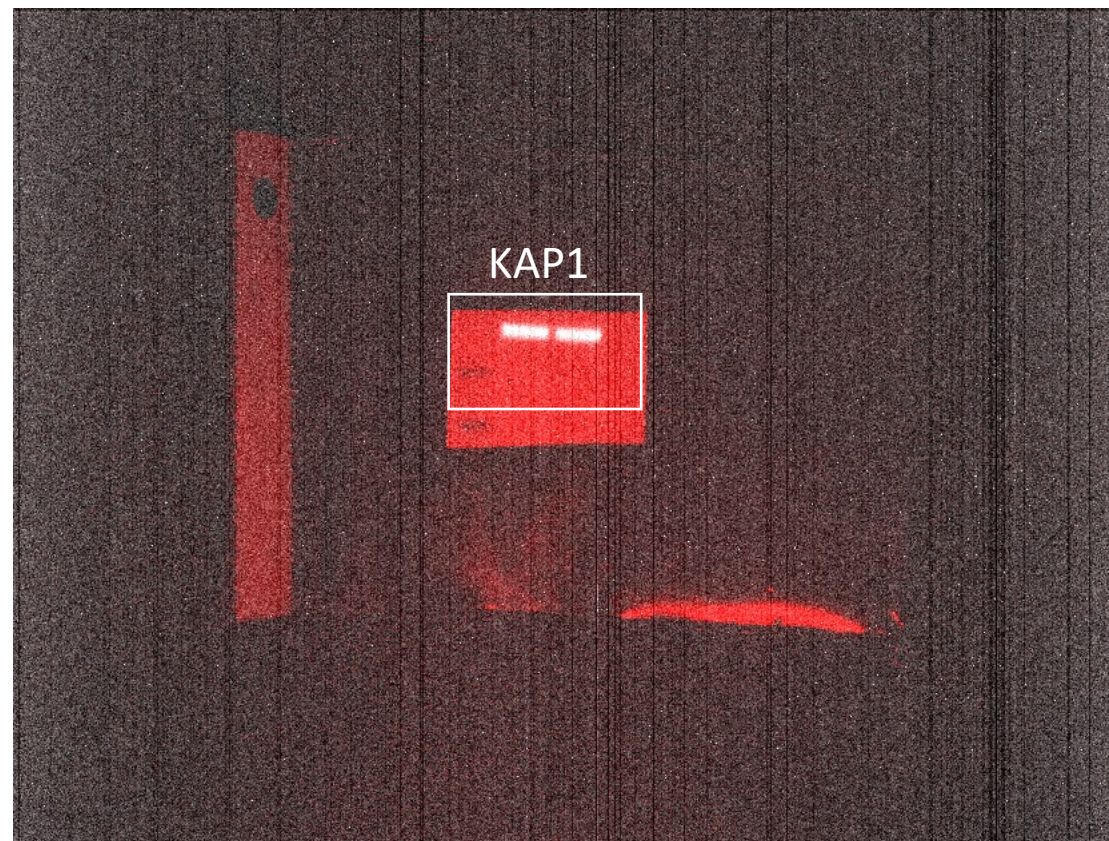

Supplement: Figure 2—figure supplement 1—source data 1. [file elife-74700-fig2-figsupp1-data1.pdf]

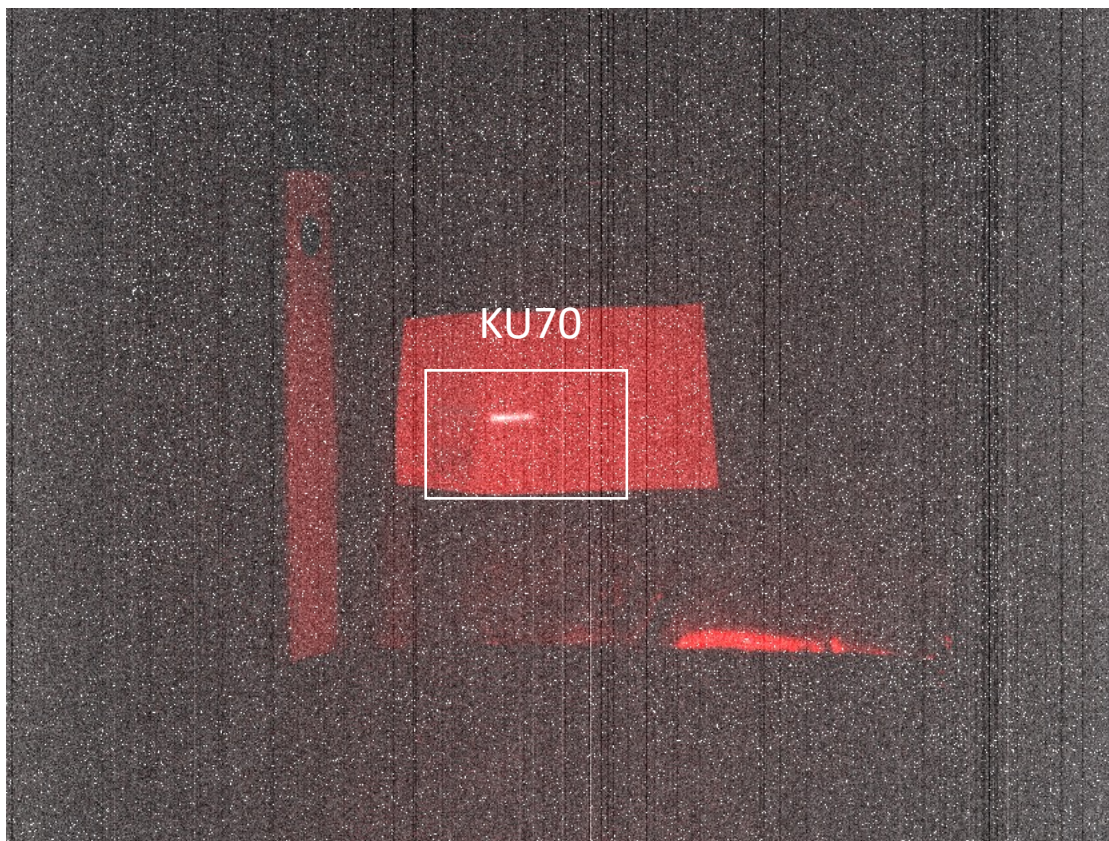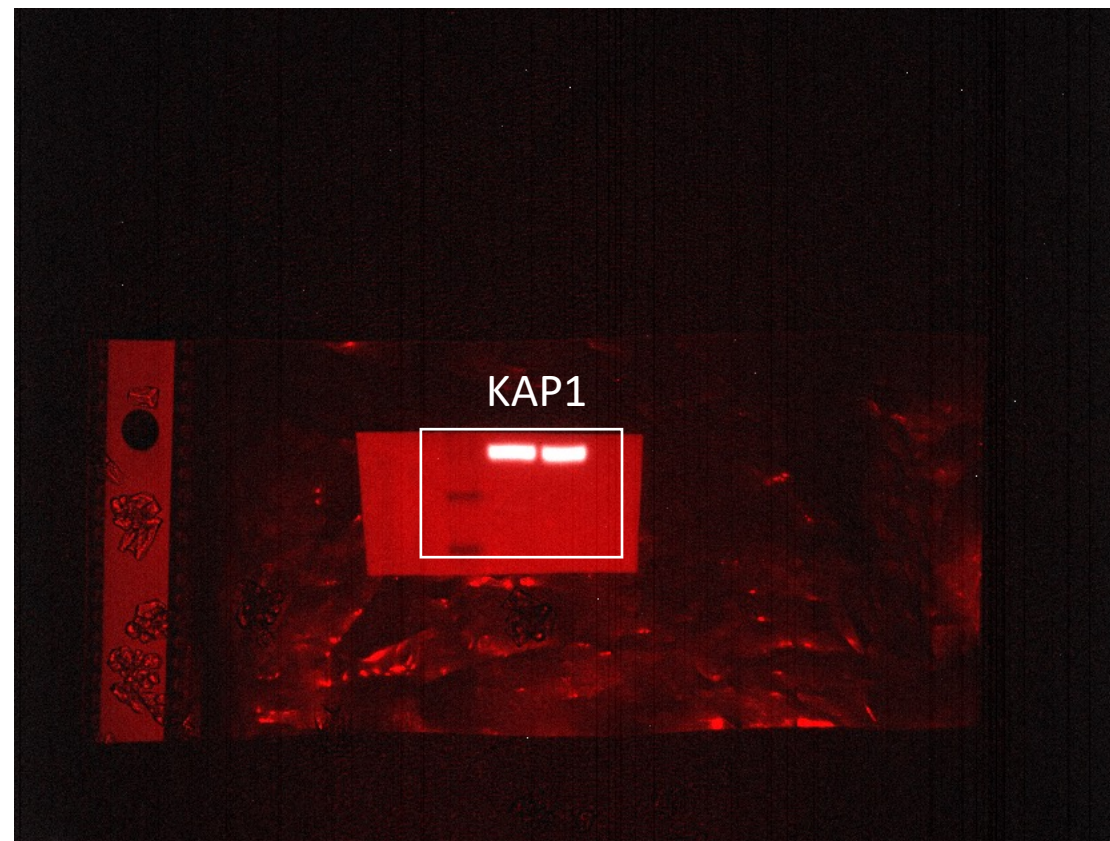

Supplement: Figure 3—figure supplement 1—source data 1. [file elife-74700-fig3-figsupp1-data1.pdf]

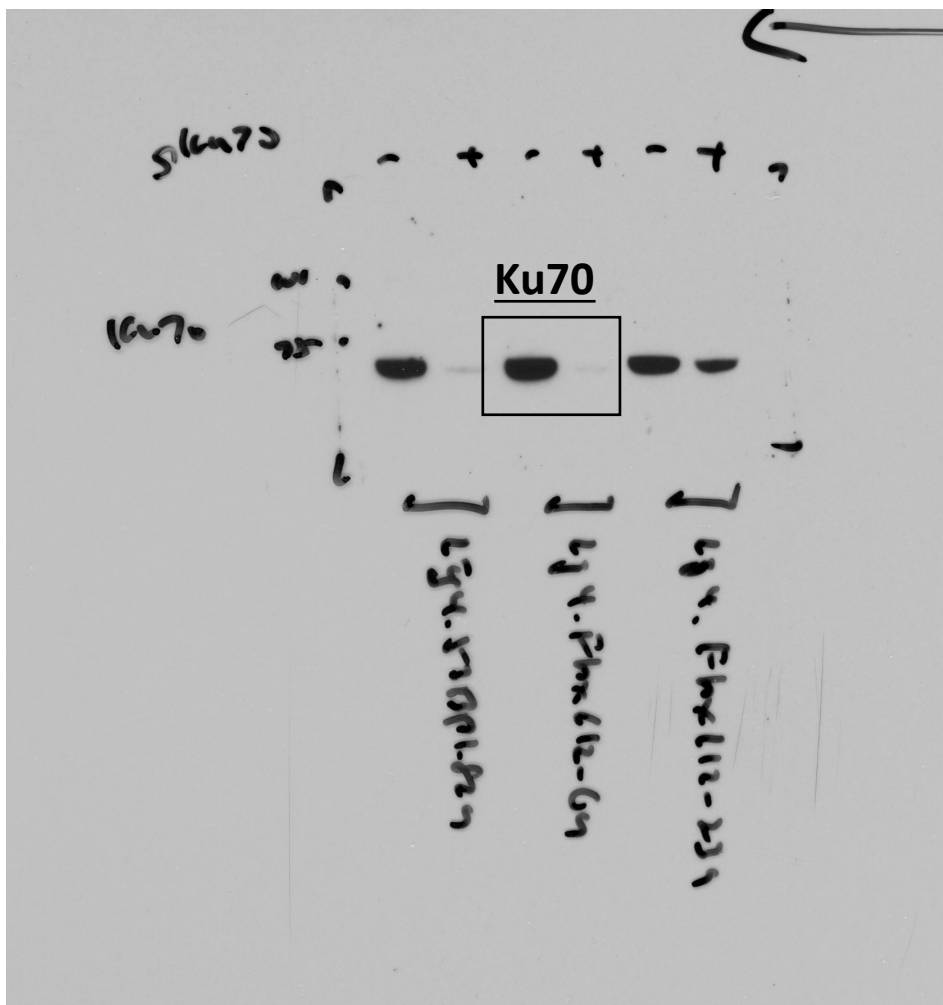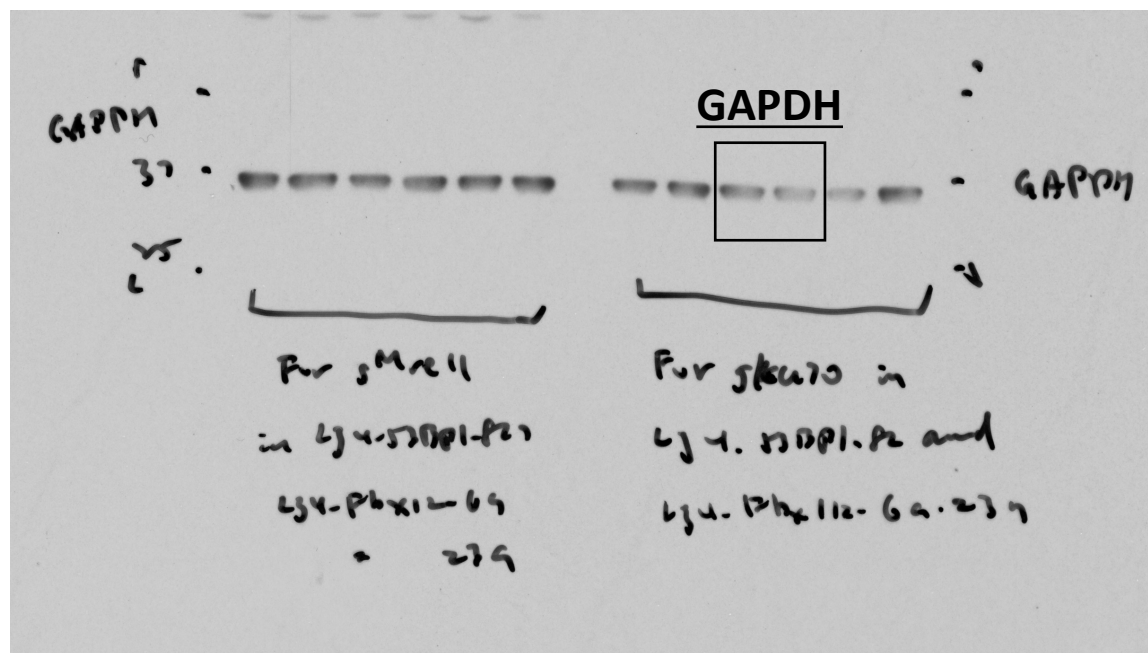

Supplement: Figure 3—figure supplement 1—source data 2. [file elife-74700-fig3-figsupp1-data2.pdf]
